# Comparative Genome Analysis Reveals Accumulation of Single-Nucleotide Repeats in Pathogenic *Escherichia* Lineages

**DOI:** 10.3390/cimb44020034

**Published:** 2022-01-20

**Authors:** Koji Ishiya, Nobutaka Nakashima

**Affiliations:** Bioproduction Research Institute, National Institute of Advance Industrial Science and Technology, Sapporo 062-8517, Japan; koji.ishiya@aist.go.jp

**Keywords:** comparative genomics, *Escherichia*, homopolymeric tracts, single-nucleotide repeats, transcriptional regulation, pathogenic lineages

## Abstract

Homopolymeric tracts (HPTs) can lead to phase variation and DNA replication slippage, driving adaptation to environmental changes and evolution of genes and genomes. However, there is limited information on HPTs in *Escherichia*; therefore, we conducted a comprehensive cross-strain search for HPTs in *Escherichia* genomes. We determined the HPT genomic distribution and identified a pattern of high-frequency HPT localization in pathogenic *Escherichia* lineages. Notably, HPTs localized near transcriptional regulatory genes. Additionally, excessive repeats accumulated in toxin-coding genes. Moreover, the genomic localization of some HPTs might be derived from exogenous DNA, such as that of bacteriophages. Altogether, our findings may prove useful for understanding the role of HPTs in *Escherichia* genomes.

## 1. Introduction

Homopolymeric tracts (HPTs), also referred to as single-nucleotide repeats, are nucleotide repeats consisting of one of the nucleotides (A, T, G, or G). HPTs are a feature of phase variation and can lead to changes in bacterial gene expression [1,2,3,4,5,6,7]. In bacteria, the ability to regulate gene expression according to environmental conditions is important for survival. Repetitive DNA sequences, such as HPTs, cause polymerase slippage during DNA replication, resulting in DNA strand expansion and contraction [8]. These slippage events can be a driving force for the evolution of genes and genomes [9,10,11]. Therefore, the genomic distribution of repetitive DNA sequences involved in acquisition mechanisms of HPTs is an important topic from an evolutionary standpoint. Although HPTs have been explored in several bacterial species, limited comprehensive analyses of HPTs have been performed within the *Escherichia* genus, which is the most widely used genus for generating genetically engineered bacteria and shows pathogenic variation among strains.

Here, we performed a comprehensive comparative genome analysis for identifying and comparing HPTs across members belonging to the genus *Escherichia*. Our results provide a comprehensive picture of the distribution of HPTs across *Escherichia* genomes and reveal their strain-specific profiles.

## 2. Materials and Methods

Our study mainly consisted of the following three steps to determine the status of HPTs in *Escherichia* strains: data collection, data sorting, and HPT detection (Figure S10).

### 2.1. Data Collection

All published *Escherichia* genome sequences were obtained from the National Center for Biotechnology Information (NCBI) GenBank (https://www.ncbi.nlm.nih.gov/genbank/; last accessed on 21 August 2020) and RefSeq (https://www.ncbi.nlm.nih.gov/refseq/; last accessed on 21 August 2020) using the NCBI Genome Downloading Scripts ver. 0.2.12 (https://github.com/kblin/ncbi-genome-download; last accessed on 12 April 2020). To unify the completeness of the genome assembly, downloaded sequences were limited to those at the complete assembly level. As a result, 1346 assembly sequences (FASTA format) were collected along with the coding nucleotide sequences (FASTA format) and annotation tables (GFF). These data were grouped into 140 taxonomies according to the Taxonomy IDs in NCBI (https://www.ncbi.nlm.nih.gov/taxonomy; last accessed on 21 August 2020). The complete assembly genomes of the *Propionibacterium* and *Helicobacter* were also collected for interspecies comparison in their HPTs. The collected assembly sequences are summarized in Table S5.

### 2.2. Detection of HPTs

In this study, an HPT was defined as a region with six or more nucleotide repeats, according to a previous study on *Propionibacterium acnes* [11]. We explored HPTs across the collected assembly sequences using SeqKit, a toolkit for FASTA/Q file manipulation [12]. To investigate the nucleotide bias in the HPTs, the lengths of A/T/G/C repeats in the HPTs were measured by an in-house Python script. The percentages of genomic features with ≥6 repeats were calculated with the pandas package ver. 1.0.3 [13] in Python. To statistically evaluate the differences in the number of repeats among the features, multiple comparisons were performed using Tukey’s honestly significant difference test [14] (familywise error rate α = 0.05) with the statsmodels package ver. 0.12.2 [15] in Python.

To further investigate the accumulation of HPTs on phage-derived sequences of pathogenic strains, we used PHASTER web server (http://phaster.ca/; last accessed on 2 November 2021) [16] to infer bacteriophage-derived regions of their genomes. The detection of HPTs was also performed on the inferred regions. These results are summarized in Table S4.

### 2.3. Analysis of Intragenic HPTs

Intragenic HPTs were identified using the coding regions of DNA or RNA. All detected intragenic HPTs were subjected to annotation validation based on the GFF files. To reduce the effect of incomplete genome assembly, we focused on genes containing intragenic HPTs in all strains. Furthermore, to evaluate variation in intragenic HPTs, genes with significantly different HPTs across strains were identified according to the coefficient of variation of repeat lengths [17]. To explore strain-specific patterns in intragenic HPTs, a cluster analysis based on Euclidean distance was performed using the seaborn package ver. 0.11.1 (https://seaborn.pydata.org/; last accessed on 5 October 2020) [18] in Python.

### 2.4. Analysis of Intergenic HPTs

To elucidate the characteristic patterns of intergenic HPTs appearing between genes, we identified the nearest neighbor sequences of the detected HPTs. Thus, a BED file was prepared containing the genomic coordinates of 150 bases on both sides of intergenic HPTs, and HPT flanking regions (FASTA format) were extracted. With these FASTA files as input queries, a homology search was performed using the BLASTX [19] algorithm of DIAMOND ver. 0.9.24.125 [20] against all protein-coding sequences for *Escherichia*. Query sequences with more than 90% homology and an *E*-value < 1 × 10^−9^ were classified as the nearest neighbor genes of the intergenic HPTs. In addition, the frequency of occurrence of genes located in the neighboring HPT region was calculated for all strains. A cluster analysis based on Euclidean distance was conducted for determining strain-specific features in the neighboring genes.

### 2.5. Phylogenetic Analysis

To clarify taxonomic profiles of strains and HPT localization patterns, we performed a phylogenetic analysis including all 140 strains of the genus *Escherichia*. The samples used for this phylogenetic analysis are summarized in Table S6. Firstly, core genes among all strains were identified using Roary ver. 3.11.2 [21]; next, a maximum likelihood phylogenetic tree was constructed from these core gene sequences using iqtree ver. 1.6.12 [22]. Finally, the tree was visualized using iTOL (https://itol.embl.de/; last accessed on 10 October 2020) [23].

### 2.6. Correlation Analysis between Pathogenic Factors and HPTs

We used the PathoFact pipeline ver. 1.0 [24] for HPT-accumulating (O-157) and non-accumulating (K12) strains to search their genomes for potentially pathogenic factors, coding bacterial toxin-related ORFs. To investigate the association between pathogenicity and HPT accumulation, correlation analysis was performed on the predicted number of predicted pathogenic ORFs and the observed number of HPTs in their genomes. These results are summarized in Figure S9.

## 3. Results and Discussion

HPTs have been identified in the genomes of several prokaryote species [2,3,4,5,7,25,26,27,28]. However, the genomic localization pattern of HPTs in *Escherichia* strains, including common species in biotechnology or clinical pathogens, is not well understood. Therefore, we performed a comprehensive survey of 1346 genomes from all 140 *Escherichia* strains and found a total of 14,335,513 HPTs with six or more nucleotides. The data for each nucleotide are shown in Table 1. Analysis of the nucleotide bias in the HPTs showed that *Escherichia* had a high proportion of A and T repeats relative to C and G repeats, with an (A + T)/(G + C) ratio of 11.60 (Table 1; Figure S1). Although this result may be partially due to poly-T tracts in Rho-independent terminators [11], the bias in this ratio may also result from their genomic nucleotide composition. Indeed, in a supplemental analysis of the genomes of other species, the (A + T)/(G + C) ratios of HPTs in *Propionibacterium* and *Helicobacter* were 0.05 and 10.0, respectively (Table S1), suggesting that the nucleotide bias of HPTs may be influenced by their genomic nucleotide composition.

**Figure 1:**
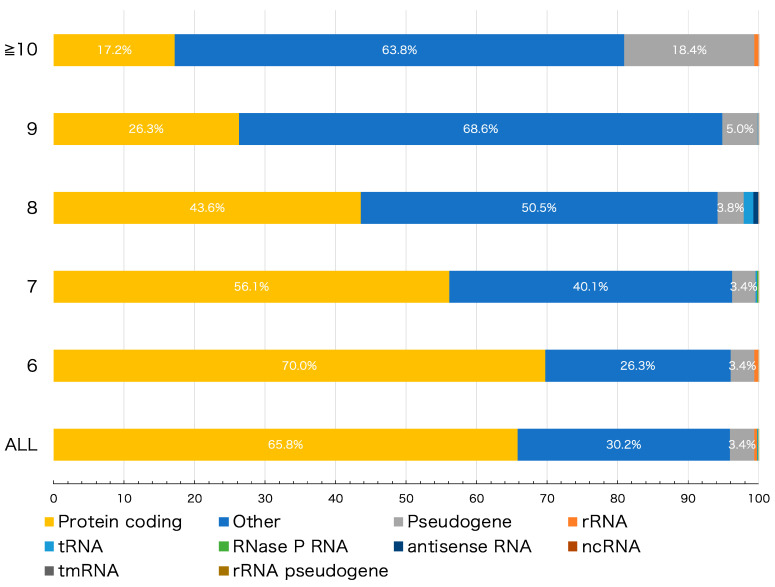
Number of homopolymeric tract (HPT) repeats in various genomic features. The bar plot shows the percentage of the genomic features to which the detected HPTs belong. “ALL” denotes all HPTs with > 6 nucleotide repeats; remaining row labels correspond to the number of nucleotide repeats in HPT. The colors correspond to different genomic features as indicated.

To investigate the role of HPTs in genomes, detected HPTs were categorized based on the following 10 genomic features: protein coding, pseudogene, ribosomal (r)RNA, transfer (t)RNA, RNase P RNA, antisense RNA, non-coding (nc)RNA, transfer-messenger (tm)RNA, rRNA pseudogene, and other intergenic HPTs located outside the coding region. The protein-coding, pseudogene, rRNA, tRNA, RNase P RNA, antisense RNA, ncRNA, tmRNA, and rRNA pseudogene features accounted for 65.78%, 3.41%, 0.44%, 0.09%, 0.03%, 0.02%, 0.02%, and 0.0003% HPTs, respectively (Figure 1, “ALL”). Similarly, detected HPTs were categorized according to the length of the repeats (Figure 1). The proportion of each genomic feature was different depending on the length of the repeats; multiple comparison tests showed a significant difference in the number of repeats in 84.4% (38/45) of the combinations among the features (adjusted *p*-value < 0.05) (Figure S2, Table S2).

**Figure 2:**
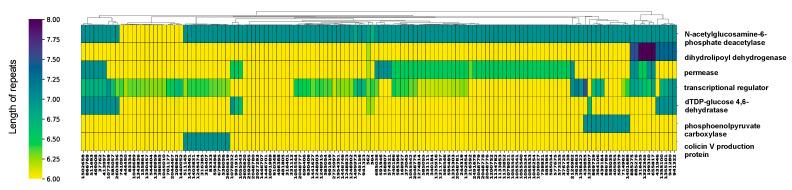
Variation in repeat length in intragenic homopolymeric tracts (HPTs) across *Escherichia* strains. The cluster map shows genes with significantly-variable intragenic HPTs in the genus *Escherichia* clustered by their repeat lengths. The horizontal axis shows the gene (protein) name, and the vertical axis shows the corresponding NCBI Taxonomy ID. The cluster map of all observed intragenic HPTs was shown in Figure S4. The color of the heatmap corresponds to the length of the repeats, with yellow and dark blue colors indicating the shortest and longest repeat lengths, respectively.

Intergenic HPTs are single-nucleotide polymorphisms present in non-coding regions and their localization patterns across the genome remain largely unexplored. The role of intragenic and intergenic HPTs might be different, and, thus, we first examined HPTs located within intragenic regions. The mean number of occurrences of intragenic HPTs was 1.93 (min = 1; max = 44.63) (Figure S3), which were identified in 119 genes in all *Escherichia* strains (Figure S4). The mean, standard deviation, minimum, and maximum values for the number of HPT repeats observed in these genes were 6.14, 0.28, 6.00, and 8.00, respectively. In contrast, for genes well conserved across strains, intragenic HPTs were mostly six-base repeats (Figure S4), indicating that the HPT repeats in non-coding regions without open reading frames (ORFs) are longer compared with those in protein/RNA-coding regions. Therefore, variation in length is small for intragenic HPTs and extension of repeat length is restricted to maintain protein functions. Among the identified genes, those with significant variation in the number of HPT repeats across the strains coded for transcriptional regulators, dihydrolipoyl dehydrogenase, permease, dTDP-glucose 4,6-dehydratase, colicin V production protein, phosphoenolpyruvate carboxylase, and *N*-acetylglucosamine-6-phosphate deacetylase (Figure 2; |Z| > 1.96, two-sided tests).

We next examined HPTs located within intergenic regions. As shown in Figure 1, the percentage of intergenic HPTs increased to more than 60 % when the number of repeats increased to 10 or more (see “Others”). A total of 9272 genes were found to reside in the nearest neighbor sequences of HPTs (Table S3). Of these, genes encoding hypothetical proteins having unknown functions showed the highest percentage, accounting for 8.91% of the total genes flanking intergenic HPTs (Figure S5). Except for genes encoding hypothetical proteins, the top 30 genes with the highest percentage are shown in Figure S6. In particular, 12 strains (taxid: 1343836, 386585, 155864, 1045010, 1330457, 544404, 444450, 1328859, 741093, 83334, 1048689, and 701177) showed a high frequency of HPTs with transcriptional regulatory genes as neighbors. Notably, most strains that showed this pattern belonged to enterohemorrhagic *Escherichia coli* strains, such as O-55 and O-157 (Figure S6). It has been reported that some HPTs with significant repeat length variation among strains are characteristically located in or near transcriptional regulatory genes, thereby affecting their function [5,7,27,28]. An alternative theory is that the differences in the number of repeats are caused by exogenous DNA from bacteriophages. In fact, pathogenic *E. coli* strains, such as O-157 Sakai, have a higher proportion of exogenous DNA than other non-pathogenic strains [29]. In addition, our analysis showed that in the O-157 Sakai strain, approximately 20% of observed HPTs were located in genomic regions derived from bacteriophages (Table S4). This result suggests that their HPTs may have been contributed, at least in part, by exogenous-derived DNA.

These changes in the number of HPT repeats would also cause a frameshift in the gene sequence, and the stop codon could cause loss of transcriptional regulation or protein function. Indeed, we found that the number of HPT repeats and percentage of pseudogenes were positively correlated (r = 0.707; Figure S7), and the number of HPT repeats tended to be higher in pseudogenes than in other genomic features (Figure 1). Mutations, including insertions and deletions of HPTs, cause frameshifts that result in multiple open reading frames that cannot be assigned a function in gene prediction.

**Figure 3:**
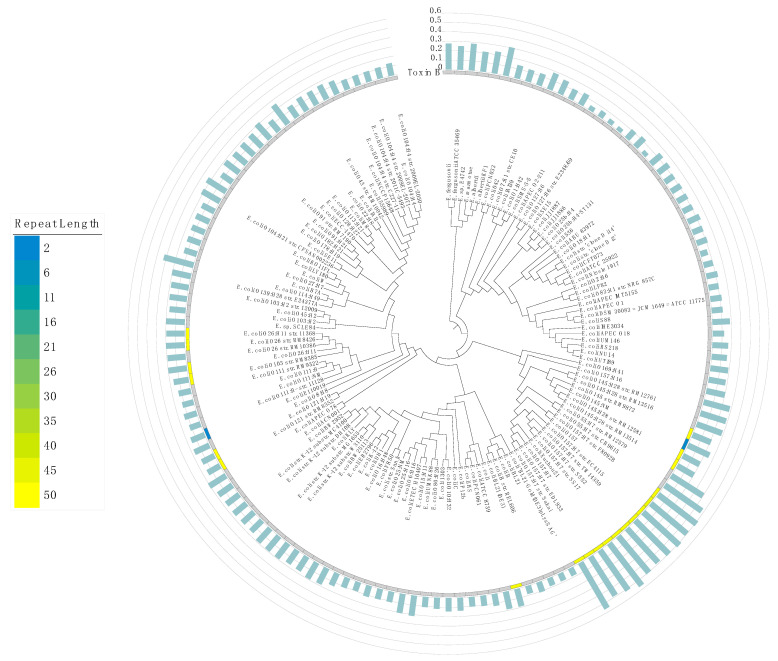
Maximum likelihood phylogenetic tree of the genus *Escherichia*. The phylogenetic tree was constructed based on the core genes in all 140 strains of the genus *Escherichia*; the NCBI Taxonomy ID was replaced by the name of each strain. The distribution of the number of HPT repeats in the toxin B gene is shown in a heatmap. Blue and yellow colors denote the smallest and largest number of repeats, respectively. The gray color indicates the loss of toxin B. The bar plot in light green shows the frequency of occurrence of transcriptional regulatory genes in intergenic HPTs (%). The axis of the bar plot is 0 to 0.6% with 0.1% intervals.

Our analysis revealed HPTs with long repeat lengths in several genes (Figure S8). In particular, the toxin B gene in some strains has long HPTs (Figure 3). These overrepresented repeats might have been derived from exogenous DNA from bacteriophages, such as the Shiga toxin gene *stx* in the O-157 strain [30,31]. In addition, we also examined the number of potentially pathogenic ORFs and HPTs among strains that differed in their pathogenicity (e.g., O-157 and K12 strains); we observed a significant positive correlation between the number of pathogenic ORFs and HPTs (Figure S9; *R* = 0.99, *p* = 1.6×10^−12^). This result suggests that the accumulation of HPTs could be associated with their pathogenic factors. Interestingly, HPTs were also observed at high frequency in O antigen genes, which have been used to classify serotypes. This suggests that the localization of HPTs contributes to O antigen variation in *Escherichia.*

In conclusion, we identified several features of HPT localization in *Escherichia* genomes, which could provide useful insights into HPT genomic diversity and plasticity.

## Figures and Tables

**Table 1:** Repeat length of the observed homopolymeric tracts.

	Count ^a^	Mean ^b^	SD ^c^	Min ^d^	Max ^e^
A	6,606,099	6.27	0.55	6.00	56
C	569,850	6.22	0.57	6.00	70
G	567,499	6.22	0.58	6.00	68
T	6,592,065	6.27	0.56	6.00	108
All	14,335,513	6.27	0.55	6.00	108

^a^ Summarized by the type of nucleotide (A/T/G/C), regardless of the length of the homopolymeric tract repeats observed. ^b^ The average homopolymeric tract repeat length. ^c^ The standard deviation in the length of the repeat. ^d^ The minimum length of repeat. ^e^ The maximum length of repeat.

## Data Availability

The datasets analyzed during the current study are available from the corresponding author on reasonable request.

## Supplementary Materials

The following are available online at https://www.mdpi.com/article/10.3390/cimb44020034/s1.
