# Supplementary material for: Comparative Genome Analysis Reveals Accumulation of Single-Nucleotide Repeats in Pathogenic Escherichia Lineages
Source: Curr Issues Mol Biol. 2022 Jan 20;44(2):498–504. doi: 10.3390/cimb44020034 (PMC8928963; doi:10.3390/cimb44020034)
Supplement: Supplementary file 1 [file cimb-44-00034-s001.zip › Figure S1.pdf]

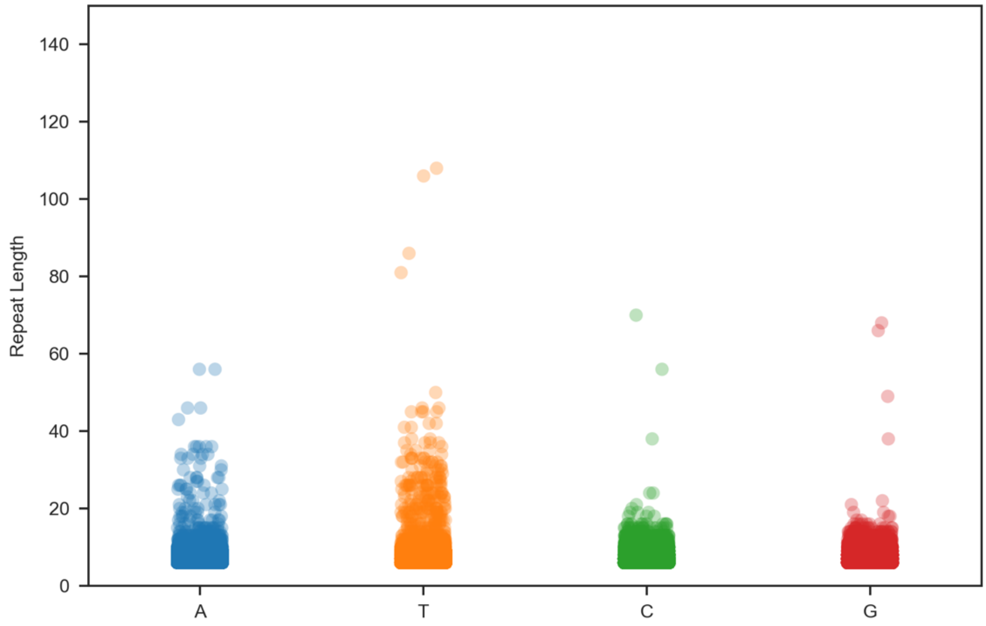

Figure S1. Number of repeats per nucleotide in homopolymeric tracts. The scatter plot shows the distribution of the number of repeats for each type of nucleotide in the homopolymeric tracts.
