# Supplementary material for: Comparative Genome Analysis Reveals Accumulation of Single-Nucleotide Repeats in Pathogenic Escherichia Lineages
Source: Curr Issues Mol Biol. 2022 Jan 20;44(2):498–504. doi: 10.3390/cimb44020034 (PMC8928963; doi:10.3390/cimb44020034)
Supplement: Supplementary file 1 [file cimb-44-00034-s001.zip › Figure S10.pdf]

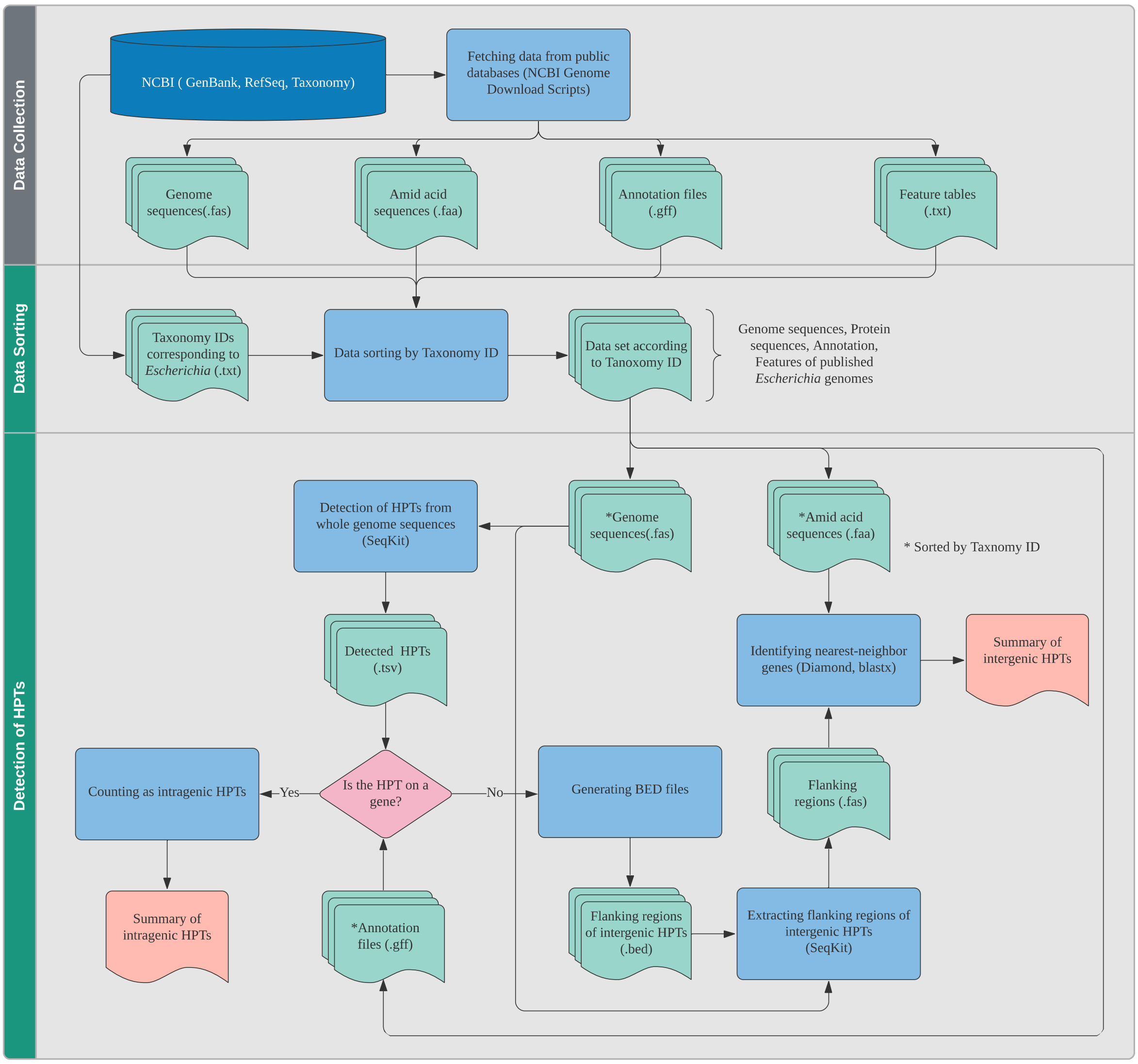

Figure S10. Schematic representation of the workflow in our study. The workflow consisted of three major steps: data collection, data sorting, and homopolymeric tract (HPT) detection. The blue cylindrical shape represents the public databases GenBank, RefSeq, and Taxonomy at NCBI. Rectangular shapes with wavy lines at the bottom indicate the input/output files; light green and orange shapes indicate multiple and single files, respectively; and the light blue rectangles denote data processing. The light pink diamond indicates a decision made in the process.
