# Supplementary material for: Comparative Genome Analysis Reveals Accumulation of Single-Nucleotide Repeats in Pathogenic Escherichia Lineages
Source: Curr Issues Mol Biol. 2022 Jan 20;44(2):498–504. doi: 10.3390/cimb44020034 (PMC8928963; doi:10.3390/cimb44020034)
Supplement: Supplementary file 1 [file cimb-44-00034-s001.zip › Figure S2.pdf]

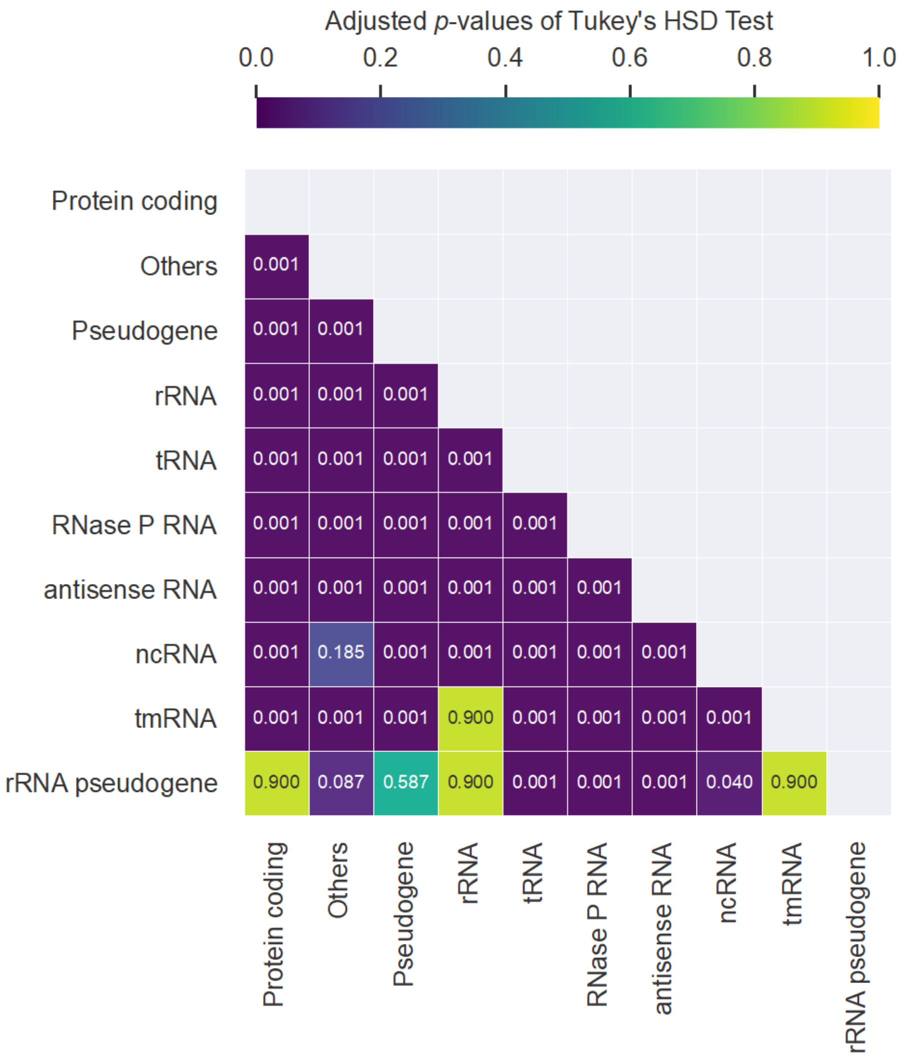


**Figure S2.** Multiple comparisons of homopolymeric tract repeat lengths between different genomic features. Multiple comparison tests were performed using Tukey's honestly significant difference test ($\alpha$ = 0.05). The colors correspond to the adjusted *p*-values. Dark blue represenst closer to 0, whereas yellow represent closer to 1.
