# Supplementary material for: Comparative Genome Analysis Reveals Accumulation of Single-Nucleotide Repeats in Pathogenic Escherichia Lineages
Source: Curr Issues Mol Biol. 2022 Jan 20;44(2):498–504. doi: 10.3390/cimb44020034 (PMC8928963; doi:10.3390/cimb44020034)
Supplement: Supplementary file 1 [file cimb-44-00034-s001.zip › Figure S4.pdf]

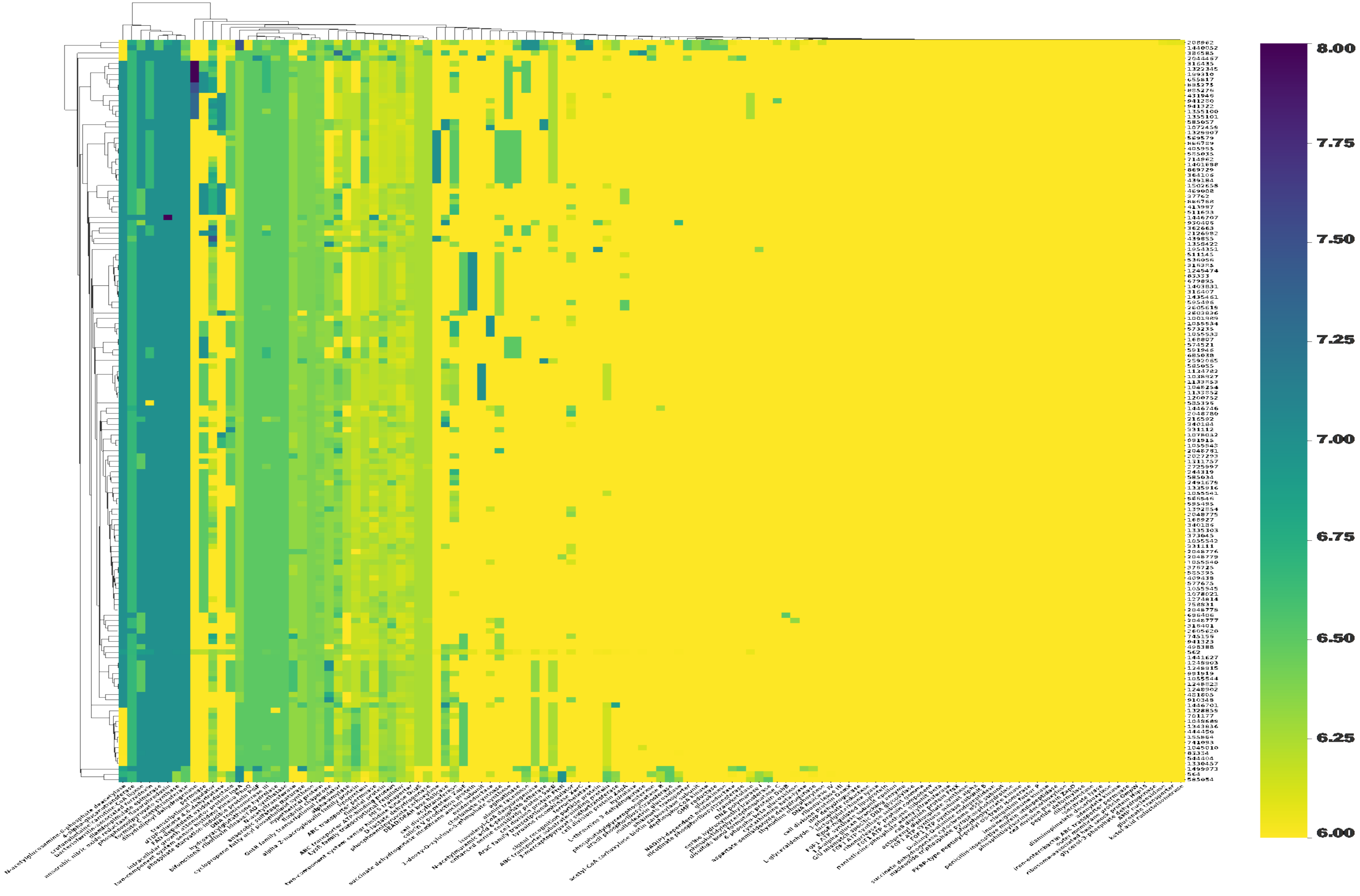

Figure S4. Variation in repeat length in intragenic homopolymeric tracts (HPTs) for genes well conserved across *Escherichia* strains. The horizontal axis shows the gene (protein) name and the vertical axis shows the corresponding NCBI Taxonomy ID. The color of the heatmap corresponds to the length of the repeats, with yellow and dark blue colors indicating the shortest and longest repeat lengths, respectively.
