# Supplementary material for: Comparative Genome Analysis Reveals Accumulation of Single-Nucleotide Repeats in Pathogenic Escherichia Lineages
Source: Curr Issues Mol Biol. 2022 Jan 20;44(2):498–504. doi: 10.3390/cimb44020034 (PMC8928963; doi:10.3390/cimb44020034)
Supplement: Supplementary file 1 [file cimb-44-00034-s001.zip › Figure S5.pdf]

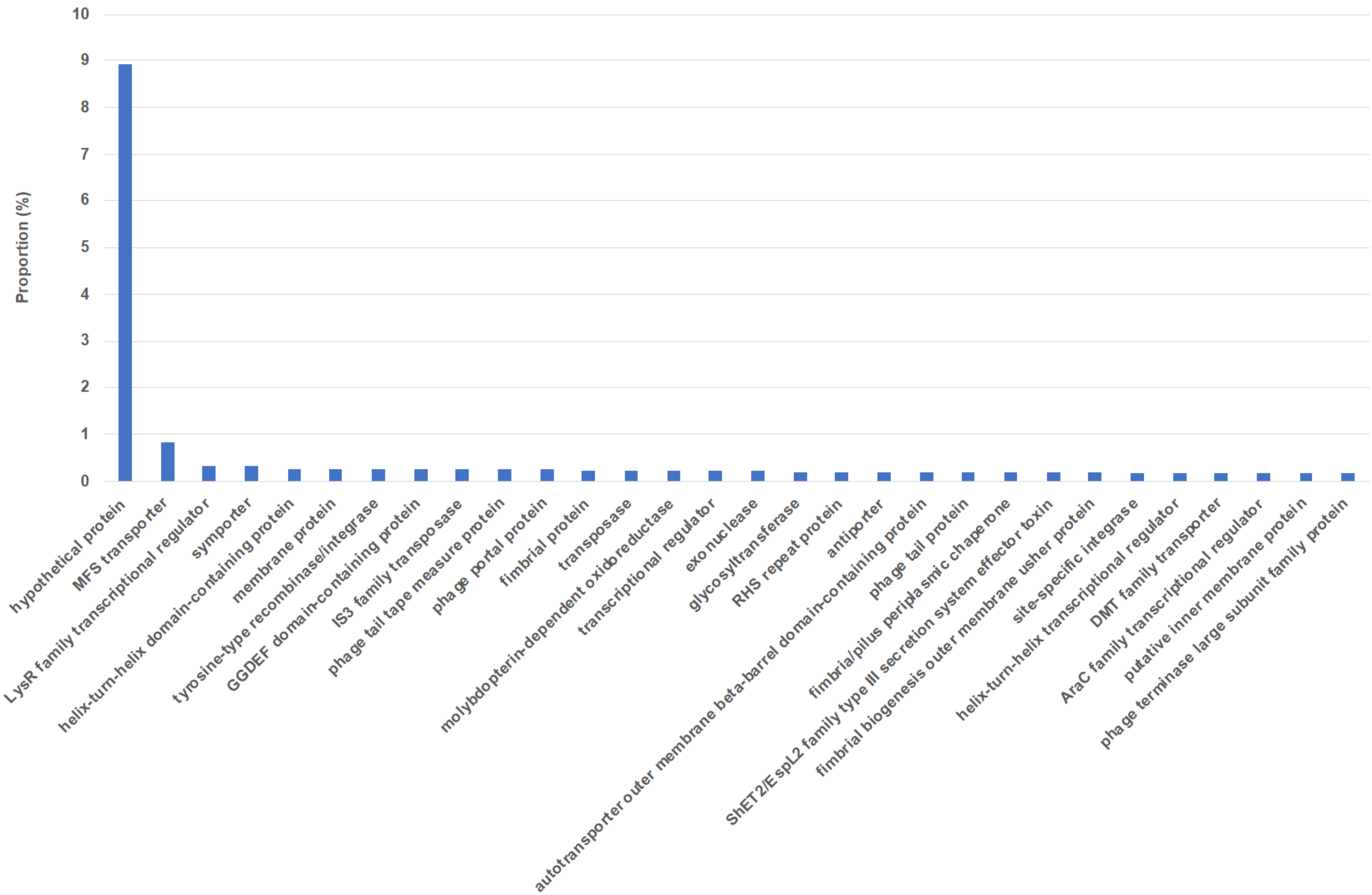

Figure S5. Genes flanking intergenic homopolymeric tracts (HPTs). The horizontal axis presents the top 30 genes located at high frequency in the neighboring areas of intergenic HPTs. The vertical axis shows the percentage of intergenic HPTs.
