# Supplementary material for: Comparative Genome Analysis Reveals Accumulation of Single-Nucleotide Repeats in Pathogenic Escherichia Lineages
Source: Curr Issues Mol Biol. 2022 Jan 20;44(2):498–504. doi: 10.3390/cimb44020034 (PMC8928963; doi:10.3390/cimb44020034)
Supplement: Supplementary file 1 [file cimb-44-00034-s001.zip › Figure S6.pdf]

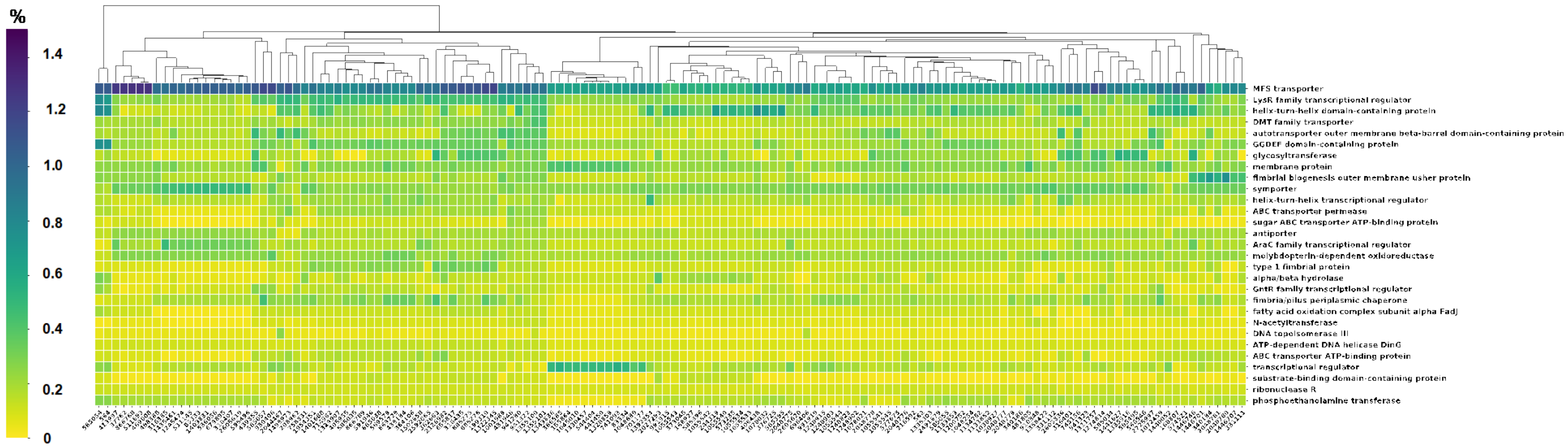

Figure S6. Taxonomic profile of intergenic homopolymeric tracts (HPTs). The heatmap represent a clustering diagram for 29 genes neighboring the intergenic HPTs shown in Figure 3, excluding genes encoding hypothetical proteins with unknown annotation. The color of the heatmap corresponds the percentage of intergenic HPTs with yellow and dark blue indicating lower and higher percentage, respectively.
