# Supplementary material for: Comparative Genome Analysis Reveals Accumulation of Single-Nucleotide Repeats in Pathogenic Escherichia Lineages
Source: Curr Issues Mol Biol. 2022 Jan 20;44(2):498–504. doi: 10.3390/cimb44020034 (PMC8928963; doi:10.3390/cimb44020034)
Supplement: Supplementary file 1 [file cimb-44-00034-s001.zip › Figure S8.pdf]

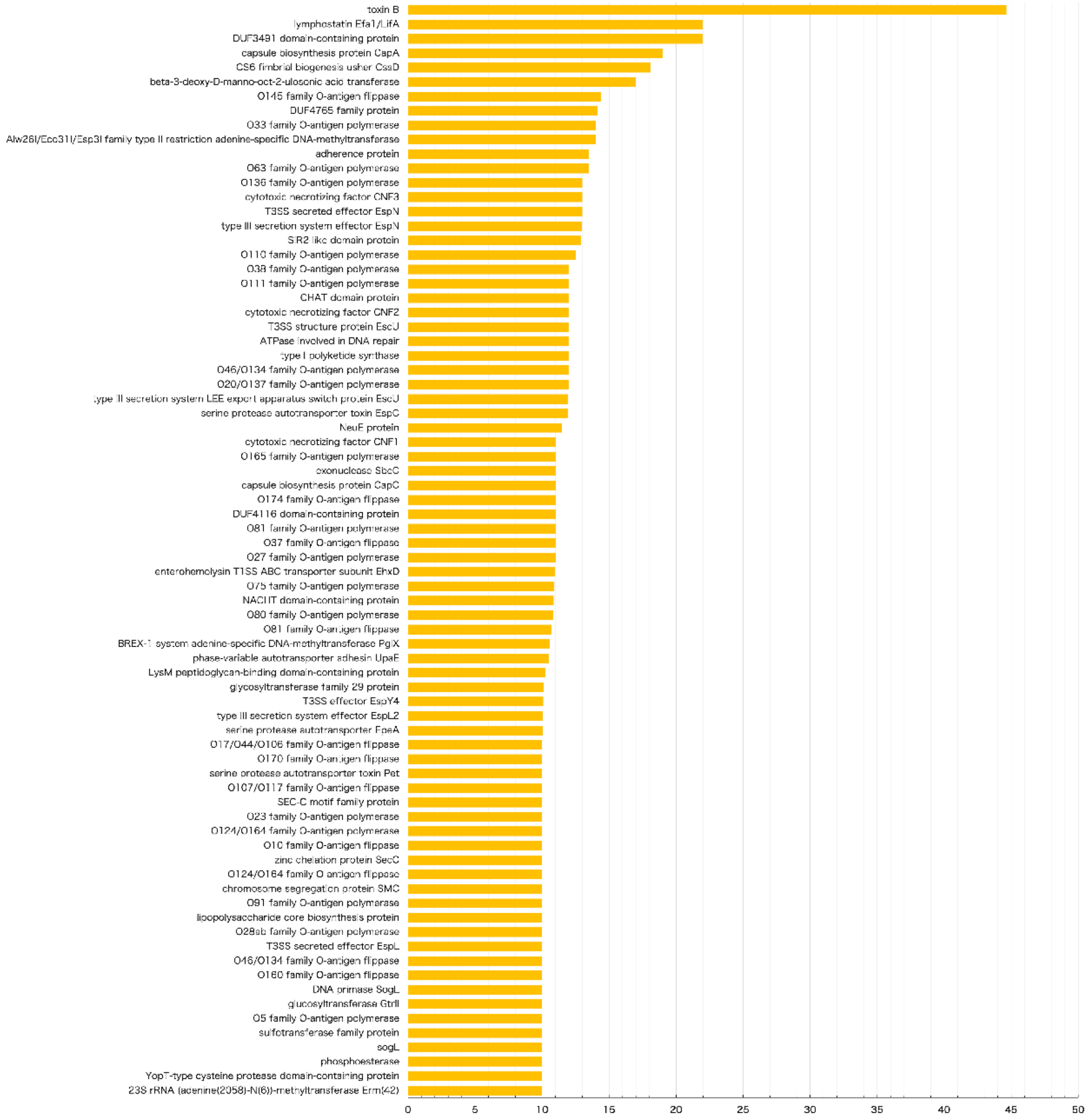

Figure S8. Genes in which homopolymeric tracts (HPTs) appear at a frequency of >10 relative to the frequency of HPTs in all annotated genes. The X and Y axes show the number of occurrences of HPTs (mean value) and gene name, respectively.
