# Supplementary material for: Comparative Genome Analysis Reveals Accumulation of Single-Nucleotide Repeats in Pathogenic Escherichia Lineages
Source: Curr Issues Mol Biol. 2022 Jan 20;44(2):498–504. doi: 10.3390/cimb44020034 (PMC8928963; doi:10.3390/cimb44020034)
Supplement: Supplementary file 1 [file cimb-44-00034-s001.zip › Figure S9.pdf]

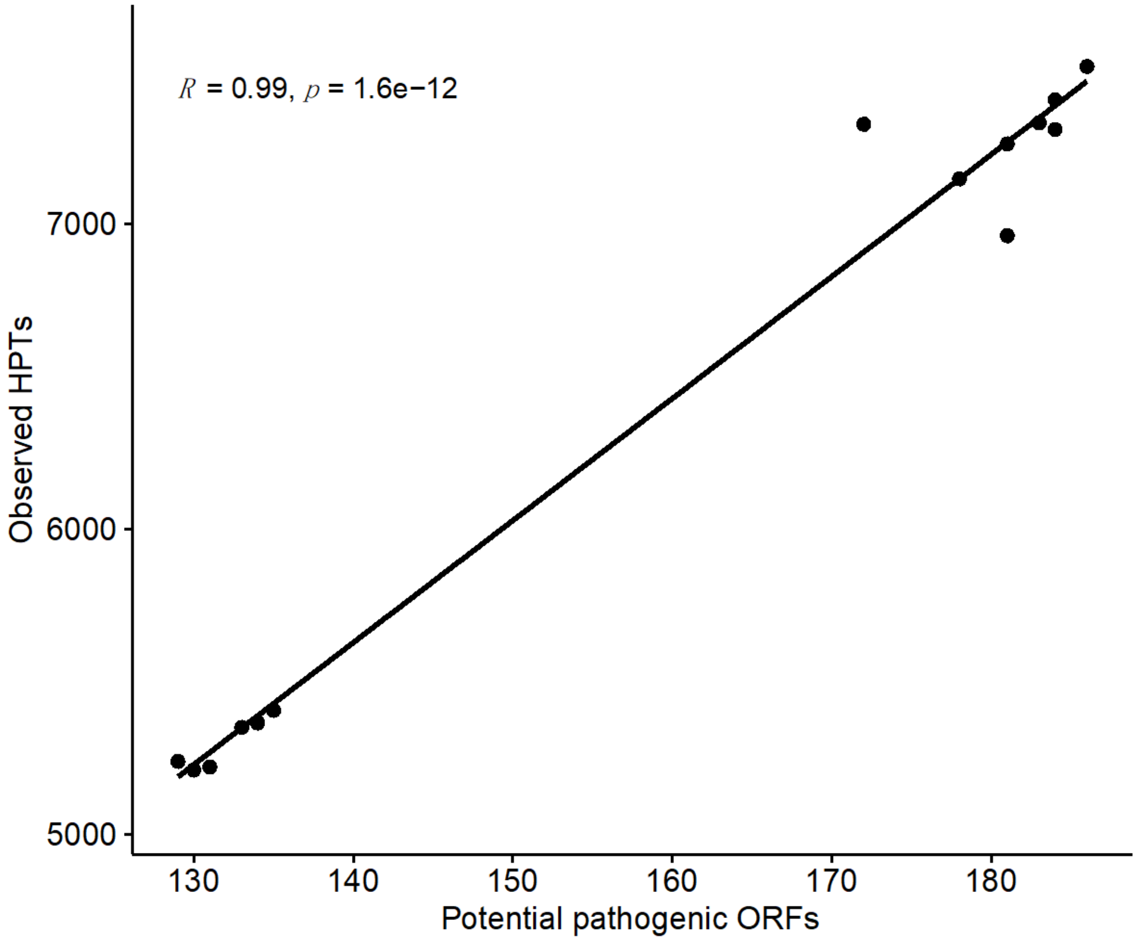

Figure S9. Scatter plots in the number of potential pathogenic ORFs and the number of HPTs. These pathogenic ORFs were estimated by the PathoFact pipeline. These ORFs contain the coding regions of bacterial toxin genes. *R* and *p* indicate the Pearson's correlation coefficient and the *p*-value in the uncorrelated test, respectively.
