# Supplementary figures and images for: Comparative Genome Analysis Reveals Accumulation of Single-Nucleotide Repeats in Pathogenic Escherichia Lineages
Source: Curr Issues Mol Biol. 2022 Jan 20;44(2):498–504. doi: 10.3390/cimb44020034 (PMC8928963; doi:10.3390/cimb44020034)

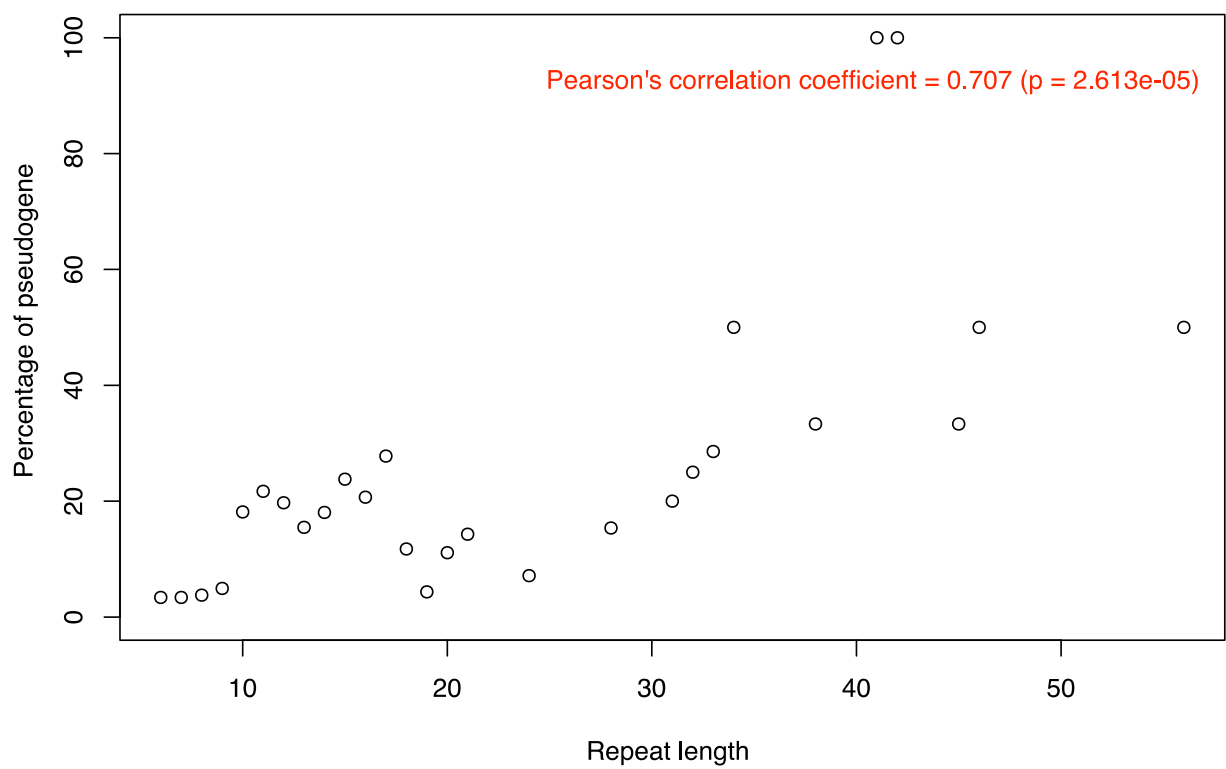

**Figure S7.** Scatter plot of homopolymeric tract repeat lengths in pseudogenes.

Supplement: Supplementary file 1 [file cimb-44-00034-s001.zip › Figure S7.pdf]
